# Sepse, Fibrilação Atrial e Envelhecimento: Uma Associação Perigosa

**DOI:** 10.36660/abc.20230095

**Published:** 2023-03-22

**Affiliations:** 1 Universidade de São Paulo Faculdade de Ciências Farmacêuticas de Ribeirão Preto Ribeirão Preto SP Brasil Universidade de São Paulo - Faculdade de Ciências Farmacêuticas de Ribeirão Preto, Ribeirão Preto, SP – Brasil; 2 Associação Ribeirãopretana de Ensino, Pesquisa e Assistência ao Hipertenso - AREPAH Ribeirão Preto SP Brasil Associação Ribeirãopretana de Ensino, Pesquisa e Assistência ao Hipertenso - AREPAH, Ribeirão Preto, SP – Brasil; 3 Faculdade IPEMED de Ciências Médicas Belo Horizonte MG Brasil Faculdade IPEMED de Ciências Médicas, Belo Horizonte, MG – Brasil; 4 Michigan State University Pharmacology and Toxicology Dept. College of Osteopathic Medicine MI EUA College of Osteopathic Medicine, Pharmacology and Toxicology Dept., Michigan State University, MI – EUA

**Keywords:** Fibrilação Atrial, Sepse, Idoso, Mortalidade, Arritmias Cardíacas, Mortalidade Hospitalar

A fibrilação atrial (FA) é um distúrbio comum do ritmo cardíaco frequentemente identificado durante a progressão da sepse.

Sua prevalência aumenta na população geral com a idade, chegando a 18% nos idosos acima de 85 anos.^1^

Pacientes com FA apresentam pior qualidade de vida, maior risco de desenvolver insuficiência cardíaca (IC), acidente vascular cerebral, declínio cognitivo, depressão, maior número de internações e maior taxa de mortalidade do que aqueles sem FA.^2^

A sepse é a principal causa de morte em unidades de terapia intensiva (UTI). Segundo o *Latin American Institute for Sepsis Studies* (ILAS), a sepse afeta de 20 a 30 milhões de pessoas em todo o mundo.^3^ No Brasil, 1000 pessoas morrem por hora, e a taxa de ocupação da UTI é de 25%, com taxa de mortalidade de 28%-54%.^3^ Segundo o Estudo Epidemiológico de Sepse no Brasil (BASES), um estudo de coorte multicêntrico realizado em 5 UTIs de hospitais públicos e privados brasileiros, a incidência de sepse é de 57,9 por 1.000 pacientes-dia (IC 95% 51,5-65,3).^4^

Revisões sistemáticas, com metanálise, avaliaram 225.841 pacientes com fibrilação atrial de início recente (NOAF) na presença de sepse. Uma correlação positiva foi encontrada entre sepse, mau prognóstico e aumento da mortalidade. Em comparação com pacientes sem FA, maior taxa de recorrência de FA, maior tempo de internação em UTI/hospitais e, consequentemente, aumento de custos.^5^^-^^8^ Uma revisão sistemática recente, com metanálise desenvolvida por Corica et al.,^9^ mostrou que a NOAF é comumente encontrada durante a sepse, estando presente em 1 em cada 7 indivíduos. Pacientes com FA têm maior risco de eventos adversos durante a sepse e necessitam de terapias específicas.^9^

Devido ao aumento da gravidade dos pacientes, o aparecimento da FA pode ser um divisor de águas/ponto de viragem entre a vida e a morte.

O estudo AFSEMA evidenciou que a FA aumentou a mortalidade hospitalar dos pacientes em uma taxa de 34,1%.^10^ O estudo revelou fatores de risco (FR) para FA, como insuficiência cardíaca com FA prévia e achados ecocardiográficos com aumento do átrio esquerdo e redução da fração de ejeção do ventrículo esquerdo (ICFEr). Além disso, valores mais elevados do escore Sequential Organ Failure Assessment (SOFA) e maior risco de arritmias, além de níveis elevados de proteína C-reativa, reforçam a hipótese de que a inflamação é um gatilho essencial para a FA.^10^

Embora a FA esteja associada a pacientes com condições clínicas de alta gravidade durante a doença crítica, essa arritmia parece aumentar a gravidade da sepse, per se, como variável independente. A predição precoce de FA durante a sepse permitiria testar intervenções na UTI para preveni-la e evitar complicações.^11^

Outra revisão sistemática, com metanálise, analisou FRs para NOAF em pacientes com sepse. Os dados revelaram que as arritmias atriais de início recente estão associadas à sepse aguda e não são FR para FA associada à comunidade.^12^ Outros fatores de risco relevantes para FA que devem ser considerados são doenças cardíacas pré-existentes (doença arterial coronariana, aterosclerose subclínica, valvulopatias, cardiomiopatias, obesidade, diabetes, hipertensão, apneia do sono, hipertireoidismo subclínico, dislipidemia, tabagismo, alcoolismo, sedentarismo), fatores relacionados à IC ou ICFEr, doença pulmonar obstrutiva crônica, doença renal crônica e a gravidade da doença sepse per se. Além disso, o envelhecimento masculino, a raça e os fatores genéticos são considerados FR independentes.^1^^,^^10^^,^^13^

A etiologia da FA é multifatorial e pode ser desencadeada por fatores que perturbam a condução elétrica cardíaca normal, como hipocalemia, hipomagnesemia e hipovolemia, além de alterações na atividade parassimpática e simpática, levando focos atriais a desenvolver automaticidade anormal, ação autossustentável potenciais ou circuitos reentrantes.^14^

A sepse é um estado inflamatório sistêmico e a FA pode se desenvolver por infiltração direta de células inflamatórias e dano oxidativo aos miócitos atriais. Os agentes vasoativos frequentemente utilizados na UTI, como a dopamina e a norepinefrina, podem levar ao aumento das descargas atriais ectópicas, desencadeando o aparecimento da FA.^14^

Embora a FA induzida por doença crítica também siga o desenvolvimento de um substrato atrial suscetível combinado com um evento desencadeador, fatores específicos que contribuem para o substrato arritmogênico podem diferir da FA adquirida na comunidade.

A perda aguda da sístole atrial e a frequência ventricular rápida que caracterizam o início da FA levam à diminuição do débito cardíaco e ao comprometimento hemodinâmico, piorando o quadro clínico e o prognóstico do paciente.

Exames de imagem demonstram que pacientes com sepse apresentam dilatação biventricular com disfunção sistólica e diastólica, sem relação com a perfusão coronariana. Sugere-se que as citocinas circulantes e a produção local de fatores depressores cardíacos sejam as causas subjacentes dessa “cardiomiopatia séptica”.^15^

A NOAF pode ser uma resposta cardíaca disfuncional à infecção com fortes implicações prognósticas conforme descrito no estudo AFSEMA^10^ e pode representar uma disfunção orgânica subestimada definidora de sepse, principalmente em idosos.

Identificar, diagnosticar e tratar a sepse em idosos permanece um desafio devido às suas manifestações atípicas. As alterações fisiológicas do envelhecimento e as múltiplas comorbidades dificultam o diagnóstico e o tratamento precoce.^5^

Os médicos devem estar cientes de que o NOAF na sepse não é apenas uma arritmia temporária, mas um marcador de mau prognóstico e deve ser tratado adequadamente.^15^
