# Supplementary material for: Sepse, Fibrilação Atrial e Envelhecimento: Uma Associação Perigosa
Source: Arq Bras Cardiol. 2023 Mar 22;120(3):e20230095. [Article in Portuguese] doi: 10.36660/abc.20230095 (PMC10399966; doi:10.36660/abc.20230095)
Supplement: Translation

# Translation: Sepsis, Atrial Fibrillation, and Aging: A Dangerous Association

**Affiliations:** 1 Ribeirão Preto SP Brazil Universidade de São Paulo - Faculdade de Ciências Farmacêuticas de Ribeirão Preto, Ribeirão Preto, SP – Brazil; 2 Ribeirão Preto SP Brazil Associação Ribeirãopretana de Ensino, Pesquisa e Assistência ao Hipertenso - AREPAH, Ribeirão Preto, SP – Brazil; 3 Belo Horizonte MG Brazil Faculdade IPEMED de Ciências Médicas, Belo Horizonte, MG – Brazil; 4 MI USA College of Osteopathic Medicine, Pharmacology and Toxicology Dept., Michigan State University, MI – USA

**Keywords:** Atrial Fibrillation, Sepsis, Mortality, Aged, Arrhythmias, cardiac, Hospital Mortality

Atrial fibrillation (AF) is a common heart rhythm disorder often identified during sepsis's progression.

Its prevalence increases in the general population with age, reaching 18% in the elderly over 85 years old.^1^

Patients with AF present a poor quality of life, a higher risk of developing heart failure (HF), stroke, cognitive decline, depression, higher hospitalizations, and a higher mortality rate than those without AF.^2^

Sepsis is the leading cause of death in intensive care units (ICU). According to the Latin American Institute for Sepsis Studies (ILAS), sepsis affects 20 to 30 million people worldwide.^3^In Brazil, 1000 people die per hour, and the ICU occupancy rate is 25%, with a mortality rate of 28 %-54%.^3^According to the Brazilian Sepsis Epidemiological Study (BASES), a multicenter cohort study conducted in 5 ICUs from public and private Brazilian hospitals, the incidence of sepsis is 57.9 per 1000 patient-days (95% CI 51.5-65.3).^4^

Systematic reviews, with meta-analysis, have evaluated 225,841 patients with new-onset atrial fibrillation (NOAF) in the presence of sepsis. A positive correlation was found between sepsis, poor prognosis, and increased mortality. Compared to patients without AF, higher FA recurrence rate, a longer length of stay in ICU/hospitals, and consequently increased costs.^5^^-^^8^A recent systematic review, with meta-analysis developed by Corica et al.,^9^showed that NOAF is commonly found during sepsis, being present in 1 in 7 individuals. Patients with AF are at higher risk of adverse events during sepsis and need specific therapies.^9^

Due to the increased severity of patients, the onset of AF can be a watershed /turning point between life and death.

The AFSEMA study evidenced AF as increasing hospital mortality of patients at a rate of 34.1%.^10^The study revealed risk factors (RF) for AF, such as heart failure with previous AF and echocardiographic findings with enlarged left atrium and reduced left ventricular ejection fraction (HFrEF). Moreover, higher values of the Sequential Organ Failure Assessment (SOFA) score and increased risk of arrhythmias, in addition to high levels of C-reactive protein, support the hypothesis that inflammation is an essential trigger for AF.^10^

Although AF is associated with patients with high-severity clinical conditions during critical illness, this arrhythmia seems to increase the severity of the sepsis, per se, as an independent variable. Early prediction of AF during sepsis would allow testing interventions in the ICU to prevent it and avoid complications.^11^

Another systematic review, with a meta-analysis, analyzed RFs for NOAF in patients with sepsis. The data revealed that new-onset atrial arrhythmias are associated with acute sepsis and are not RF for community-associated AF.^12^Other relevant risk factors for AF that must be considered are pre-existing heart diseases (coronary artery disease, subclinical atherosclerosis, valvulopathies, cardiomyopathies, obesity, diabetes, hypertension, sleep apnea, subclinical hyperthyroidism, dyslipidemia, smoking, alcoholism, sedentary lifestyle), factors related to HF or HFrEF, chronic obstructive pulmonary disease, chronic kidney disease and the severity of the disease sepsis *per se* . In addition, aging male gender, race, and genetic factors are considered independent RF.^1 , 10 , 13^

The etiology of AF is multifactorial and may be triggered by factors that disturb normal cardiac electrical conduction, such as hypokalemia, hypomagnesemia, and hypovolemia, in addition to changes in parasympathetic and sympathetic activity, leading atrial foci to develop abnormal automaticity, self-sustaining action potentials or reentrant circuits.^14^

Sepsis is a systemic inflammatory state, and AF can develop by direct infiltration of inflammatory cells and oxidative damage to atrial myocytes. Vasoactive agents often used in the ICU, such as Dopamine and Norepinephrine, can lead to an increase in ectopic atrial discharges, triggering the onset of AF.^14^

Although critical illness-induced AF also follows the development of a susceptible atrial substrate combined with a triggering event, specific factors that contribute to the arrhythmogenic substrate may differ from community-acquired AF.

The acute loss of atrial systole and the rapid ventricular rate that characterize the onset of AF lead to a decrease in cardiac output and hemodynamic impairment, worsening the patient's clinical condition and prognosis.

Imaging tests demonstrate that patients with sepsis have biventricular dilatation with systolic and diastolic dysfunction, which are unrelated to coronary perfusion. It is suggested that circulating cytokines and local production of cardiac depressant factors are the underlying causes of this “septic cardiomyopathy”.^15^

NOAF may be a dysfunctional cardiac response to infection with strong prognostic implications as described in the AFSEMA Study^10^and may represent an underestimated sepsis-defining organ dysfunction, mainly in the elderly.

Identifying, diagnosing, and treating sepsis in the elderly remains challenging due to its atypical manifestations. Physiological alterations of aging and multiple comorbidities make early diagnosis and treatment difficult.^5^

Physicians should be aware that NOAF in sepsis is not just a temporary arrhythmia but a marker of poor prognosis and should be treated appropriately.^15^
